# Prevalence and availability of specialty dental care sites for children covered by Medicaid and CHIP in the United States

**DOI:** 10.3389/froh.2026.1887868

**Published:** 2026-09-01

**Authors:** JonMichael Cope, Aaron David, Chase Otto, Inder Sehgal

**Affiliations:** College of Dental Medicine, Kansas City University, Joplin, MO, United States.

**Keywords:** CHIP, endodontic, InsureKidsNow, Medicaid, pediatric

## Abstract

**Introduction:**

Access to dental care is a critical aspect of children's healthcare. For these reasons, Medicaid and Children's Health Insurance Program (CHIP), administered by states, cover medically necessary services. Specialty providers can enroll and participate in state or state-contracted plans, but availability varies widely. Our research objective was to evaluate the availability of non-general dentist specialists in endodontics, oral and maxillofacial surgery, orthodontics/dentofacial orthopedics on the InsureKidsNow website for Medicaid/CHIP enrollees, on a state-by-state basis, and to compare these with pediatric dental availability.

**Methods:**

This is an observational, cross-sectional descriptive study. InsureKidsNow.gov was searched to identify and compare available and participating endodontists, oral and maxillofacial surgeons, and orthodontic/dentofacial orthopedic surgeons with pediatric dentists. The percentages of specialists listed on InsureKidsNow were compared with the respective percentages as a proportion of the state's dental workforce. A subanalysis of 25 states that listed a single plan was conducted to determine the patient-to-listed location ratio for each specialist category. For the largest benefit program, the combined percentage of the three specialties was graphed by state along with pediatric dentists. Data were grouped by whether they accepted new pediatric patients and by the total number of specialty locations.

**Results:**

Children enrolled in Medicaid have very limited or no access to endodontists, oral and maxillofacial surgeons, or orthodontic/dentofacial orthopedic surgeons in half of the U.S. states. Thirteen states showed no availability of any non-primary care specialist for new participants, with the shortage being most pronounced for endodontic services. Among states using only one benefit plan, 12 of 25 showed no availability for new endodontic patients. In contrast to the non-primary care specialties, pediatric dentists were open to new patients in most states.

**Conclusions:**

Despite improved use of dental services among children, those covered by public plans have limited access to one or more specialty provider types in most states. This limited access is most pronounced for endodontic services and may result from relatively low availability and/or participation of endodontic locations.

## Introduction

Access to dental care is recognized by the Center for Medicaid & Medicare Services (CMS) as a critical aspect of child healthcare (1). This access reduces the overall cost and health burden (2), reduces future caries risk (3), and decreases the health equity gap (4). A 2018 study found that Medicaid cost savings associated with preventive oral healthcare, such as daily fluoride use and sealants, exceeded $7M per year (2). Medicaid and the Children's Health Insurance Program (CHIP) cover dental services such as routine check-ups, X-rays, fluoride treatments, dental sealants, and fillings (5). States are required to provide dental benefits to children covered by Medicaid and the Children's Health Insurance Program (CHIP), and the services must, at a minimum, include pain and infection relief, tooth restoration, maintenance of dental health, and medically necessary orthodontic services (6, 7). States must post their listings of participating Medicaid and CHIP dental providers on InsureKidsNow.gov (IKN) (5, 8).

The CMS established the Oral Health Initiative in 2010 to expand access to preventive dental care. Programmatically, states are required to provide medically necessary dental services to children, including treatment services such as pulp therapy for permanent and primary teeth, removable prosthesis, orthodontic treatment when medically necessary to correct handicapping and other malocclusions (9), and fixed bridges and implants when determined to be medically necessary (10). However, states are not specifically required to provide access to specialty dentists who typically provide these services, such as oral and maxillofacial surgery (OMS), endodontists, and orthodontists. Because CMS requirements are focused on the child's oral health condition rather than provider-specific, some states may have gaps in access for children who require care from other specialists. The “Guide to Children's Dental Care in Medicaid” acknowledges that appropriate care may necessitate referrals to dental specialists and that inadequate specialty networks lead to unmet treatment and comprehensive care needs among eligible children (10).

Children enrolled in Medicaid have higher dental care utilization rates than uninsured children, at 43.7% and 20.4%, respectively. The percentage of Medicaid-enrolled children and adolescents (ages 1–20) who receive dental care has continued to increase nationwide, from 29.3% in 2000 to 52.3% in 2019 (11). The increased rate of pediatric dental utilization has been driven primarily by the expansion of publicly funded insurance programs (12). However, there are barriers to participation, such as low reimbursement rates, administrative burdens, and poor patient compliance (7, 13). In addition, rural communities face particular challenges in ensuring access to oral healthcare. These communities have low provider-to-population ratios and an inadequate number of dentists who accept Medicaid (7).

The literature suggests a need for specialty practitioners to care for children. A recent survey reported that a significant portion of pediatric dentists refer most endodontic cases (14), yet child patients face challenges with these referrals because fewer endodontists than pediatric dentists accept public payer insurance (12, 14). Recent data describe participation in primary dental care, defined as dentists who provided at least one oral examination or fluoride treatment to at least one Medicaid beneficiary under 21 years of age in 2018 (11). However, to our knowledge, recent research has not evaluated the availability of, not just participation, of non-general practitioner specialists, for Medicaid/CHIP enrollees on a state-by-state basis.

Our research objective was to address this gap. The IKN website is for children and adolescents up to 19 years of age. Using the “Find a Dentist” tool on IKN, we identified the percentage of all listed specialist locations and compared this with the percentage of those specialists in the state. The IKN specialty categories include Endodontics, OMS, and orthodontics/dentofacial orthopedics (ODO). As a reference category, we included Pediatrics (a specialty considered to be both primary care and specialty) location participation. We identified both practitioner locations accepting new patients and the total number of locations listed.

## Methods

### Data collection procedures

This was an observational, cross-sectional descriptive study to assess the availability of non-pediatric specialist locations in IKN Medicaid/CHIP benefit plans. The non-pediatric specialty categories listed in IKN are endodontics, OMS, and ODO. Pediatric dentistry serves as the reference category (15). The search criteria were: “No” for “Does your child have special health care needs?” and specifying each state for “Dentist Location.” Data were accessed from the live IKN database between December 1 and December 16, 2025. This study did not involve human subjects; institutional review board approval was not applicable because the data sources are publicly available.

### Statistical methods

The percentages for each practice location category were averaged (with zero-availability states included in the reported means), and the categories of locations accepting new patients and categories of all participating locations were separately compared using the Kruskal–Wallis test followed by *post-hoc* Dunn's test (16–18). The IKN locator tool limits search results to 1,000, and this limit was reached in 15 states, usually for general dentistry. Because the denominator for the specialty percentages is the sum of all dental categories, the calculated percentages in these 15 states are overestimated. To determine whether this overestimation biased the results, a separate sensitivity analysis was performed. For this assessment, a reference dataset consisting of the percentages of specialties and pediatric locations from all 50 states was compared with datasets that excluded states in which at least one dental category reached the 1,000-location limit. For practice categories accepting new patients, 5 states were excluded; for categories of all participating locations, 15 states were excluded. To test the contribution of the excluded states to the sensitivity of the reference results, the reference and excluded datasets were compared using a *t*-test (for the same categories) and the Kruskal–Wallis *H*-test mean rank scores (to determine potential Medicaid access differences among the practice categories). IKN specialty location category descriptive statistics, Kruskal–Wallis *H*-test mean rank scores for reference and excluded datasets, and significant differences between practice categories were summarized (Table 1). To quantify the size of the differences between endodontic categories and the OMS, ODO, and pediatric categories, the effect size (Cohen's *d*) was calculated (19, 20). Effect sizes ±0.2 were interpreted as small, ±0.3–0.5 as medium, and ±0.8+ as large.

**Table 1:** Averages and mean rank scores (Kruskal–Wallis test) of U.S. state specialty dental location categories and pediatric dental locations listed in IKN.

Location category as a % of listings accepting new patients						
IKN practice category	For data all (*N* = 50)			For data excluded (*N* = 45)		
	Average % of total locations ± SD	Kruskal–Wallis *H* test mean rank score	Cohen’s *d* (95% CI) vs. endodontics	Average % of total locations ± SD	Kruskal–Wallis *H* test mean rank score	Cohen’s *d* (95% CI) vs. endodontics
Endodontics	1.30 ± 1.78	62.0^a^	N/A	1.09 ± 1.37	56.6^a^	N/A
OMS	6.89 ± 6.00	103.0	1.26 (1.69–0.83)	6.64 ± 6.29	90.7	1.22 (1.73–0.71)
ODO	7.11 ± 7.14	101.0	1.12 (1.54–0.69)	7.34 ± 7.41	92.1	1.17 (1.68–0.66)
Pediatrics	12.81 ± 9.31	136.0*	1.72 (2.18–1.26)	12.36 ± 8.30	122.6*	1.89 (2.46–1.33)

Location category as a % of listings participating in plan						
IKN practice category	For data all (*N* = 50)			For data excluded (*N* = 35)		
	Average % of total locations ± SD	Kruskal–Wallis *H* test mean rank score	Cohen’s *d* (95% CI) vs. endodontics	Average % of total locations ± SD	Kruskal–Wallis *H* test mean rank score	Cohen’s *d* (95% CI) vs. endodontics
Endodontics	1.51 ± 2.13	53.0^a^	N/A	1.12 ± 1.84	39.9^a^	N/A
OMS	7.79 ± 6.42	106.8	1.31 (1.75–0.88)	5.67 ± 4.56	71.7	1.31 (1.82–0.79)
ODO	7.38 ± 6.76	101.1	1.17 (1.60–0.75)	6.05 ± 5.77	70.7	1.15 (1.66–0.65)
Pediatrics	14.26 ± 10.40	141.1^a^	1.70 (2.16–1.24)	12.03 ± 8.13	99.7^a^	1.85 (2.41–1.29)

^a^ Different from other groups at alpha = 0.05.

Data are presented for the percentages of specialty dental locations searched in IKN. Comparisons between categories were made using a Kruskal-Wallis Test followed by a post-hoc Dunn's test using a Bonferroni corrected alpha. Data sets shown are for the 50-state data set (Data all) and data sets excluding states that returned location numbers exceeding the search limit (Data excluded). Effects size shows IKN Practice Category vs. Endodontics Cohen's d in bold with 95% confidence intervals bracketed in italics. All effect sizes are interpreted as large (greater than ± 0.8).

**Figure 1:**
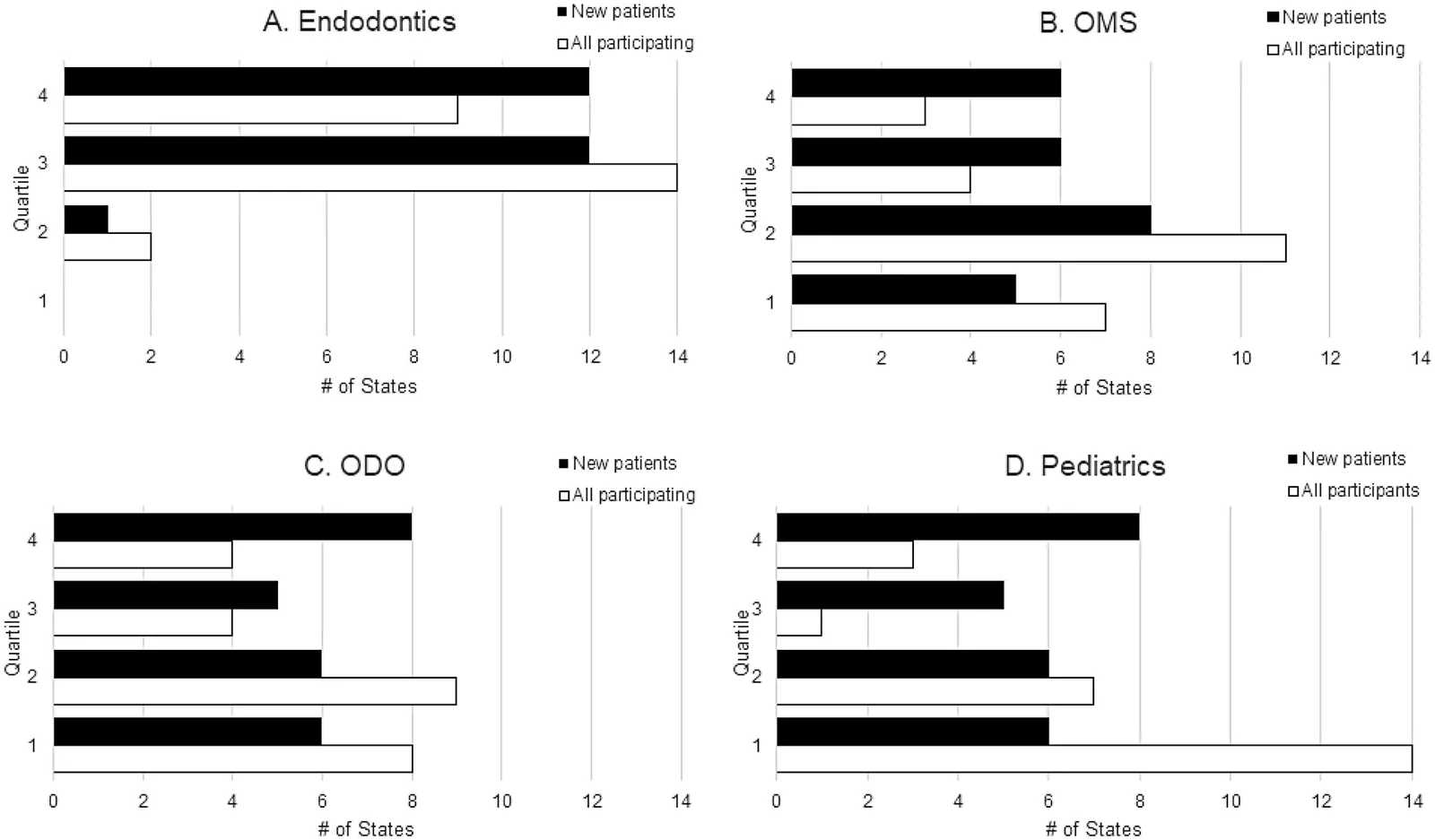
A. Endodontics; B. OMS; C. ODO; D. Pediatrics Quartile (Q) Ranges are Q4: No availability – 154,209:1 ratio; Q3: 9,638 – 143,795:1 ratio; Q2: 3,900 – 9,207:1 ratio; Q1: 614 – 3, 797:1 ratio. Black bars represent locations accepting new patients as “New patients”, white bars represent all participating locations as “All participating”.

A subanalysis was conducted for 25 states that listed a single plan, and for these states, the total number of children enrolled in Medicaid/CHIP (21) was used to determine the patient-to-listed-location ratio for each specialist category. For this subanalysis, the total potential Medicaid population was divided by the number of IKN-listed endodontic, OMS, ODO, and pediatric locations. Although this ratio is not a true caseload, it can provide a relative comparison of potential Medicaid patients to the available specialty locations. Each of the four categories for the 25 states was ranked ordinally, from no availability to the lowest patient-to-specialty location ratio. Ratios were graphed according to quartile in Figure 1. The quartiles span the minimum to maximum location participation. Quartile grouping is used by CMS to illustrate Dental Provider Participation in Medicaid and CHIP (12, 22). Instances of “No Availability” were placed after the largest numerical ratio.

States with multiple benefit plans were excluded from this aspect of the study because a single dental specialist or dental location may participate in multiple state plans, preventing accurate calculation of location-to-patient ratios. States with a single statewide benefit plan allow for a ratio to be determined for all enrollees. A Kruskal–Wallis test was performed from this subanalysis to determine whether the median ratios were the same across all location categories. Dunn's test was used for *post-hoc* analysis with the Bonferroni correction (23). Statistical summaries of these data are provided in the Results section.

### Identification of states with below average % of specialists in Medicaid/CHIP

The numbers of endodontists, OMS, and ODO (non-primary care specialties), and pediatric specialists were extracted from IKN for the largest Medicaid/CHIP benefit plan in each state. To calculate percentages, these numbers were divided by the total number of dentists listed in each plan. This percentage was calculated for both locations accepting new patients and all locations—those accepting new patients and those not accepting new patients. To compare each state with a state-specific benchmark, the specialty percentages from IKN were compared with the percentage of specialists in the state's dental workforce (24). States with lower percentages of location categories in IKN than in the specialty's state average were identified with a bullet in Table 2.

**Table 2:** States with below average % of dental specialists in Medicaid/CHIP.

U.S. state	Endodontics accepting new patients	Endodontics all providers	OMS accepting new patients	OMS all providers	ODO accepting new patients	ODO all providers	Pediatric accepting new patients	Pediatric all providers
Alabama								
Alaska	●	●						
Arizona					●	●		
Arkansas	●	●					●	●
California								
Colorado	●	●						
Connecticut	●	●						
Delaware	●	●			●	●	●	●
Florida	●	●	●	●	●	●		
Georgia					●	●		
Hawaii	●		●		●	●		
Idaho	●	●	●	●	●	●		
Illinois	●	●						
Indiana	●	●			●	●		
Iowa	●	●	●	●				
Kansas	●	●	●	●	●	●		
Kentucky	●	●	●	●	●		●	●
Louisiana	●	●	●	●	●	●		
Maine	●	●	●	●	●	●	●	●
Maryland	●	●			●	●		
Massachusetts	●	●						
Michigan	●	●			●	●		
Minnesota	●	●						
Mississippi	●	●	●	●				
Missouri	●	●	●	●	●	●	●	●
Montana	●	●	●		●		●	●
Nebraska	●	●						
Nevada	●	●			●	●		
New Hampshire	●	●	●	●				
New Jersey								
New Mexico	●	●						
New York	●	●	●		●	●	●	
North Carolina	●	●	●					
North Dakota	●	●				●		
Ohio	●	●						
Oklahoma	●	●						
Oregon	●	●	●	●	●	●	●	●
Pennsylvania	●	●						
Rhode Island	●	●			●	●		
South Carolina	●	●						
South Dakota	●	●						
Tennessee	●	●						
Texas	●	●	●	●	●	●	●	●
Utah	●	●						
Vermont	●	●			●	●		
Virginia	●	●						
Washington	●	●	●	●	●	●	●	●
West Virginia	●	●	●	●	●		●	
Wisconsin	●	●	●	●	●			
Wyoming	●	●	●		●	●	●	
Totals below state average	45	44	20	15	25	22	12	9
No participant providers listed	20	15	14	8	17	12	10	6
Participant providers >0 to <1%	9	15	1	1	2	1	0	0

States in which the percentages of IKN-listed endodontists, OMS, ODO, or pediatric dentists are below the state average for that specialty are identified by a bullet ●. Summaries show total states below average, states with no listed IKN participants for a category, and states with greater than zero but less than 1% of the category participating.

### Mapping of the percentage of dental specialists serving Medicaid/CHIP child patients in the U.S. states

**Figure 2:**
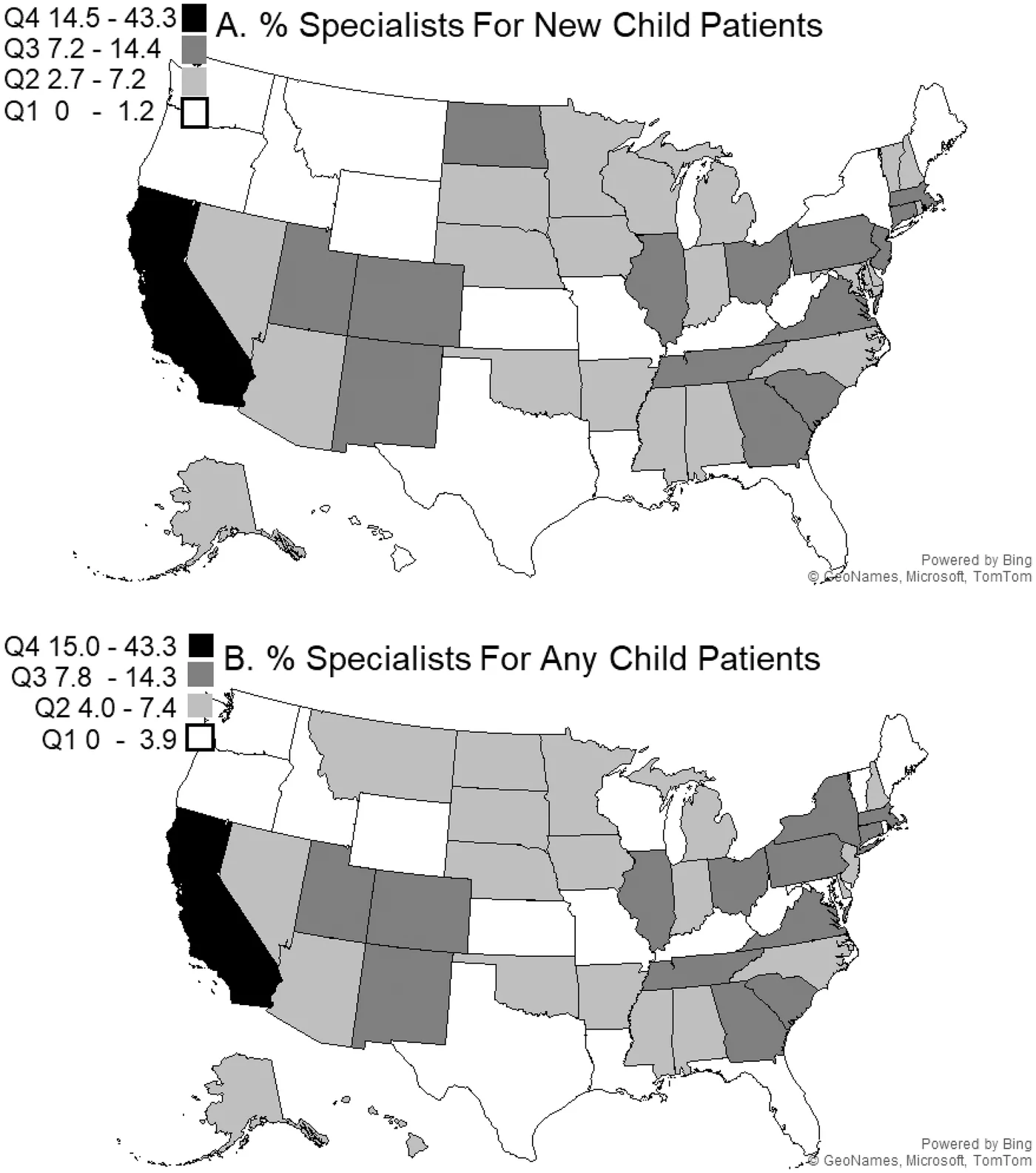

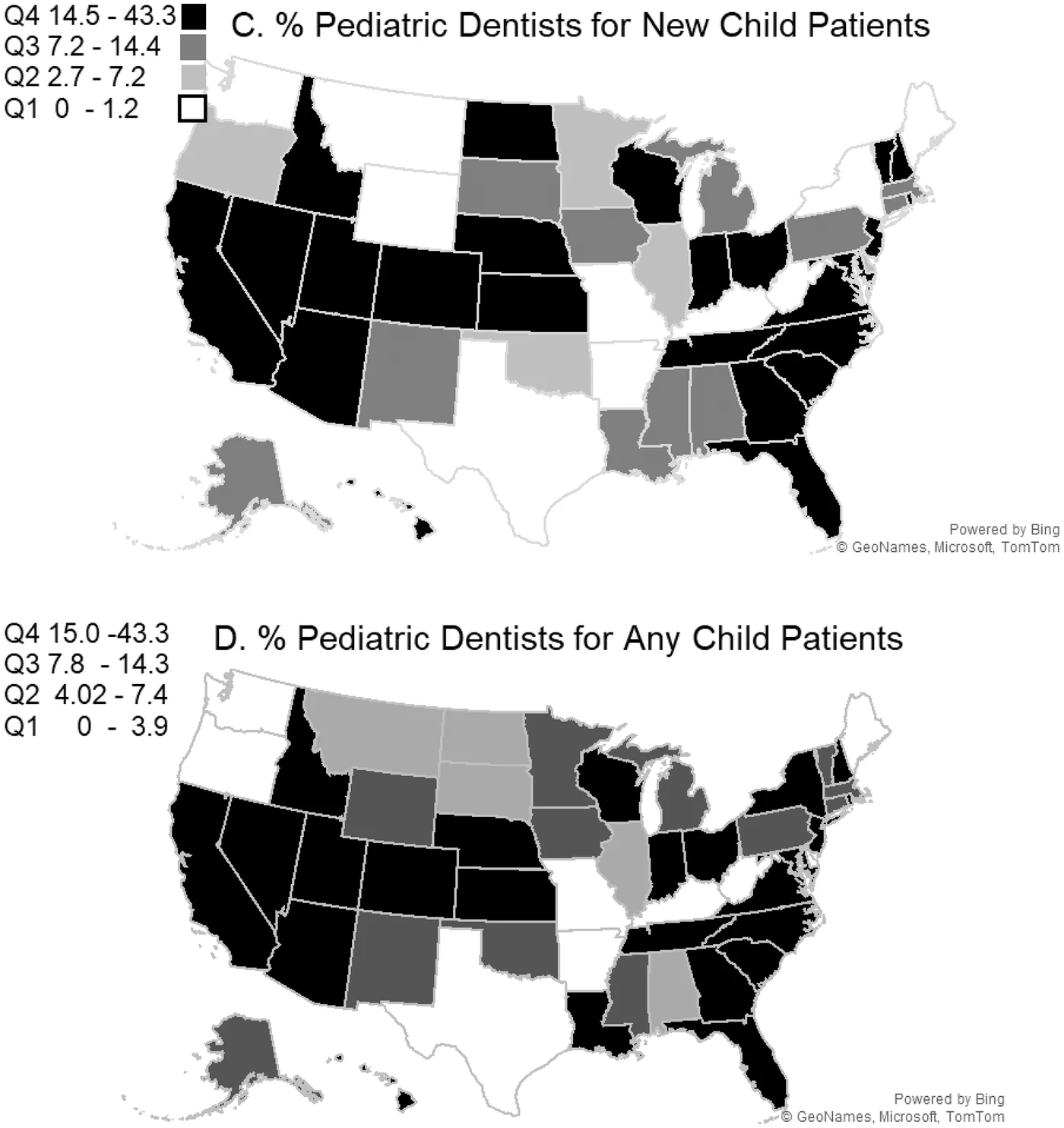
States are grouped by quartile based on the ranked percentages of non-pediatric and pediatric dental specialists participating in the state's largest benefit program. Maps show averaged endodontist, OMS, ODO listed. Panels are: Accepting new child patients **(A)** and specialists listed for any child **(B**, accepting new and not accepting new patients), pediatric dentists accepting new patients **(C)** and listed for any child patients **(D**, accepting new and not accepting new patients). Ranges for the quartiles differ between locations accepting new patients and all locations participating in the plan.

To compare non-pediatric specialties with pediatrics across all states, the individual percentages for endodontic, ODO, and OMS locations on IKN were summed and then divided by 3 to yield an average percentage for the three non-pediatric specialties. These specialty averages were then ranked alongside the averages for pediatric dental listings. The rankings for specialties were categorized as follows: specialists accepting new child patients, specialties accepting any child, pediatric dentists accepting new children, and pediatric dentists accepting any child. The new child category pairs were compared using a two-sample Mann–Whitney *U*-test (Wilcoxon rank-sum test) (25). The same comparison was performed between any child pairs. *P*-values, means, and medians are provided in the Results section. The states are illustrated on a U.S. map (Microsoft Excel 365, v.2510, Microsoft Corporation) according to quartile rank in Figure 2. The maps illustrate the percentages of specialist locations accepting new pediatric patients and all listed locations, including those not accepting new patients.

## Results

Comparison of the specialty practice categories showed that the ranked percentages of endodontic locations participating in IKN were significantly lower than those of OMS and ODO. This was the case for those accepting new patients and for all listed provider locations. The percentage of pediatric dentists was higher than those of all other groups evaluated. A comparison of the reference data for all 50 states with data excluding states that reached the 1,000-location search limit revealed no significant differences. There were no significant differences between the means of like categories (e.g., Endodontics reference vs. Endodontics excluded, etc.). Effect sizes for Endodontics compared with all OMS, ODO, and pediatric categories were interpreted as large.

Nationwide, 45 states had a percentage of endodontists accepting new patients through InsureKidsNow that was lower than the state's percentage of endodontists. Of these, 20 states indicated “no providers listed,” and another 9 had fewer than 1% of plan dentists as endodontists accepting new patients. For endodontists participating in state plans for any patients (new and current), 44 states had a percentage of providers below the state average, of which 15 had “no participants” and 15 had “less than 1%” endodontists. In contrast, a majority of each state's largest plans had a higher-than-state-average percentage of OMS locations accepting new patients, as well as a higher-than-state-average percentage of total patients in the Medicaid/CHIP plan. Of the 20 states with below-average OMS levels for new patient-accepting locations, 14 had no availability.

For ODO participation, 25 states had a lower-than-state-average percentage of locations accepting new patients, 17 of which had “no availability.” In addition, 22 states were below the state average for plan-participating ODO locations, 12 of which had “no participant providers listed.”

Pediatric specialist location participation was greater than that of endodontic, OMS, or ODO locations. The percentage of pediatric participants fell below the state average in only 12 states for locations accepting new patients and in nine states for all participant locations.

A subanalysis to determine patient-to-specialty location ratios was run across 25 states with a single Medicaid/CHIP benefit payer. Numerical ratios in these states ranged from 614:1 to 154,209:1, although there were instances of no available locations across the three defined specialties as well as pediatrics. When all location categories were ranked and grouped into quartiles, endodontic patient-to-location ratios were largely in the highest quartiles, while other categories were more uniformly distributed across the quartiles. A Kruskal–Wallis analysis was performed to determine whether median ratios were equal across all location categories. The test revealed that medians were not the same (*H* = 51.5, *p* = 5.8 × 10^−9^). *Post-hoc* analysis demonstrated a significant difference between patient-to-endodontist location ratios and the other category ratios. *P*-values for endodontists accepting new patients ranged from 3.5 × 10^−3^ to 3.5 × 10^−5^. *P*-values for all endodontists participating ranged from 2.8 × 10^−4^ to 2.6 × 10^−7^.

When each state was ranked according to the combined average IKN percentages for endodontics, OMS, ODO, and pediatrics, the findings ranged from no available or participating locations to 43.3% of of all IKN-listed locations. Thirteen states had no availability in any of the three specialties, while 10 states had no availability for pediatric dentists. For new patients, the three combined averaged specialties had a mean of 5.1% ± 4.1 and a median of 5.3% listed as available, compared with a mean of 12.8% ± 9.4 and a median of 14.3% for pediatric dentists available. A two-sample Mann–Whitney *U*-test showed *P* = 1.7 × 10^−5^ between these groups. For total participating locations, the combined specialties had a mean of 5.6% ± 3.8 and a median of 5.6%, compared with a mean of 14.3% ± 10.1 and a median of 14.2% for pediatric dentists. A Mann–Whitney *U*-test showed *P* = 3.5 × 10^−7^ between these groups. These data indicate an overall greater national availability of pediatric specialists in IKN.

Illustrated on a map of the United States by quartiles (Figure 2), states with lower percentages of listed specialty provider locations were concentrated in the upper west (Washington, Oregon, Idaho, Montana, and Wyoming) and lower Midwest to South-Central United States (Missouri, Kansas, Kentucky, Mississippi, Texas, and Louisiana). Only California's largest payer reached the largest quartile for the three specialties. The geographic distribution of pediatric specialists was similar in some aspects to that of the non-primary care specialists, with a lack of available locations in parts of the Pacific Northwest, Texas, Missouri, Arkansas, and West Virginia.

## Discussion

Using the IKN database, we assessed current participation and access for new patients to non-primary care dental specialists in endodontics, OMS, and ODO in each state and compared this participation and access with those of pediatric specialists. Pediatric dentistry is defined by the American Academy of pediatric dentistry as “an age-defined specialty that provides both primary and comprehensive preventive and therapeutic oral healthcare for infants and children through adolescence” (26). Our findings demonstrate a comparative lack of endodontists compared with OMS, ODO, and pediatric dentists participating in the state's largest Medicaid/CHIP benefit provider, especially regarding new-patient availability.

While overall availability and participation for OMS and ODO services were higher, approximately a third to a half of states had evidence of low participation and/or no listings at all. This suggests that provider participation in all three non-primary care specialties studied may be insufficient to cover patient needs in several states. The geographic locations of low dental specialty Medicaid/CHIP participation states varied; shortages were greatest in the Pacific West, the lower Midwest, and the South-Central United States. In contrast, pediatric dentists generally participate in Medicaid benefit plans at above-average levels. For states using only one benefit plan, 12 of the 25 had no availability of endodontists accepting new patients and nine had no endodontists as participants.

When comparing states using each state's largest coverage provider, the percentage of in-network endodontist locations accepting new patients was lower than the endodontist statewide average in 45 states. Twenty states had no availability of endodontists accepting new patients, and another nine had fewer than 1% of plan participants as available providers. It is likely that even provider locations listed as “accepting new patients” may not have immediately available appointments. Thirteen states also showed no availability of any non-primary care specialist for new participants. In contrast, pediatric locations were open to new patients in most states.

The reasons for the relatively low availability and participation of endodontists are unlikely to be attributable to a single factor. Nationally, endodontists represent the smallest proportion of the three specialties explored: 3.49% compared with 4.46% for OMS and 6.41% for ODO (27–30). These low numbers, coupled with inhibitory factors such as lower reimbursement rates, could contribute to reduced endodontic participation.

We considered that Medicaid or CHIP spending or reimbursement may influence specialty provider participation. Data from the Medicaid and CHIP Payment and Access Commission (MACStats) (31) on Medicaid dental benefit spending by state do not strongly correlate with the state percentages of specialists among total state dentists in this report (not shown).

Availability of specialty training programs may also influence provider participation in Medicaid and CHIP plans. These data identified 15 states with no endodontists in their largest benefit plan; eight of these states have no endodontic residency program, and three have only a single residency site (32). In contrast, pediatric dentistry residencies are present in more states and in greater overall numbers (33).

States with specialists concentrated in densely populated urban areas have limited access to care for patients living in rural regions. Although American Dental Association (ADA) health policy institute data on geographical access to care show that most states with no specialty availability still have over 80% of publicly insured children living within 15 min of a Medicaid provider, these data do not distinguish between general dentists and specialty providers (30).

This study had limitations related to data on IKN. The total number of dentist locations in a plan was calculated by summing the categories: endodontics, general dentistry, OMS, ODO, and pediatric dentistry. Because the locator tool limits search results to 1,000, totals in large plans remain undercounted, leading to higher calculated category percentages for these plans. Fifteen state plans had at least one category that reached the 1,000-location limit. In all of these states, the limit was reached for general dentistry at all participating locations (“No Preference” for Accepts New Patients). Five of the 15 states also reached the limit for general dentistry locations accepting new patients. Two states reached the limit for pediatric dentists at all locations participating, and California reached the limit for OMS and ODO at all participating locations. Even with this location limit, a sensitivity analysis of our data supports the conclusion that there is a widespread shortage of endodontists and other specialists in many states. Another limitation of the search is that the dentist locator function returns results by location (addresses), meaning that a single dentist may be listed in more than one location, or more than one dentist may be listed in just one location. This affects how locations are counted, but it does not change the calculations of specialty participation. Dental specialists may participate in several Medicaid plans; the scope of comparison was limited to the largest Medicaid benefit plan to reduce this limitation. Smaller plans may have different percentages of specialist participation; the largest plans were selected because they serve the greatest number of children. In Table 2, state specialties can fall below average either because of low numbers of specialists in the state or because existing specialists do not participate in Medicaid; our study cannot distinguish between these possibilities. Finally, this study presents only descriptive statistics and surveys current accessibility to specialty services but does not assess clinical demand for these providers.

In conclusion, while the previous literature has documented the disproportionate burden faced by publicly insured pediatric patients in accessing specialty dental care (34, 35), our study extends these findings by quantifying how this profile of state level dental location access suggests that although cost barriers and access to general or all dentist for Medicaid/CHIP children have improved, access to specialty dental locations remains unaddressed and is virtually non-existent in about half of the U.S. states. The shortage is most pronounced for endodontics.

## Data Availability

The raw data supporting the conclusions of this article will be made available by the authors, without undue reservation.
